# Bibliographical Notices

**Published:** 1841-04-17

**Authors:** 


### BIBLIOGRAPHICAL NOTICES.

*History of Embalming, and of Preparations in Anatomy, Pathology, and Natural History; including an Account of a New Process for Embalming.* By J. N. GANNAL. *Translated from the French, with Notes and Additions.* By R. HARLAN, M. D. Philadelphia, 1840.

An interesting and valuable work—containing a history of the very ancient art of preserving animal matter, with an account of the modern improvements, and of a particular process of the author's. M. Gannal's method, which has received the sanction of a committee of the Academy of Medicine of Paris, consists in the employment of a salt of alumine,—the simple sulphate is the most easily prepared, and of moderate price.

"A killogram of this salt, costing about twenty cents, dissolved in two quarts of water, is sufficient in winter to preserve a body fresh, by injection, for three months.

In order to preserve a body for a month or six weeks, it is not even necessary to inject the blood-vessels—a glyster of one quart, and the same quantity injected into the æsophagus, suffices for this limited preservation. This process is adopted at Clamart for all the dead bodies destined for dissection. The preservative power of this salt will be easily understood, if its analysis be compared with that of the double sulphate given above.

One hundred parts of simple sulphate of alumine, are composed of alumine 30, of sulphuric acid 70. This salt properly prepared, exempt from iron, commonly contains from thirty-six to forty for 0|0 of water.

The following is a table of the different densities of this salt, according to the quantity of water in which it is dissolved.

A killogram dissolved in five hundred scruples of water gives a quart of liquor which marks 32° on the areometer of Baumè.

This same quantity in a quart of water

| | |
|---|---|
| Marks, | 20° |
| In two quarts, | 17° |
| In four quarts, | 8° |
| In five quarts, | 6° |

This table is important, as it gives the composition of the different liquids of which we shall see the application.

The liquid of injection, of which we have indicated the preparation and the quantity, is sufficient during winter and moderate temperatures; but when it passes 20° it ought to be more abundant, or the solution more concentrated.

When it is intended to preserve the body for a longer period, it is necessary to neutralize the sulphuric acid, which is taken up by the addition of acetate of lead. Two hundred and fifty scruples of this salt for one killogram of the dry sulphate produces the desired effect. If the preservation is to be indefinitely prolonged, the use of the acetate of lead will have a tendency at length to blacken the epidermis. Indeed, as it is impossible to cause all the lead to disappear, the small quantity of this salt remaining in the liquid will be then decomposed by the hydro-sulphuric acid disengaged by the corpse, or rather by the sulphur which it contains, and the salt of lead is changed into sulphuret, a black insoluble powder, giving to the body all the exterior aspect of the negro."

M. Gannal's work, we need hardly say, is of great importance to the practical student of anatomy, and has interest for the general professional reader.

*A visit to thirteen Asylums for the Insane in Europe; to which are added a brief notice of similar institutions in transatlantic countries, and in the United States, and an Essay on the causes, duration, termination, and moral treatment of Insanity. With copious statistics.* By PLINY EARLE, M. D., Resident Physician of Friends' Asylum for the Insane, Frankford, near Philadelphia, Pa., &c., &c. Philadelphia, 1841.

The main portion of this pamphlet has already appeared in the American Journal of the

Medical Sciences. The author has appended to his account of the thirteen European asylums which he personally visited, such notices of others (both American and European) as he could collect from various sources, and has incorporated with the whole an essay on Insanity. The increasing interest which the subject of insanity is just now arresting in the United States, and particularly in our own immediate community, makes this pamphlet particularly well-timed; the author's remarks on the treatment of insanity are judicious, and evince familiarity with the best authorities.

It is now conceded (he says) by all who are best acquainted with the management of the insane, that the first element in their moral treatment is their removal from acquaintances and former associations. One prominent advantage in such removal is the promotion of the second element of treatment, that of withdrawing the mind from its hallucinations, and attracting it into a new current of thought. For the full accomplishment, however, of this latter object, after the removal to a suitable place has been made, the almost unremitting attention of judicious care-takers is required. New objects must be presented to the view, new incentives to the mind, and no expedient which would be likely to attract the attention and divert the thoughts must be left untried. Hence, in those institutions for the treatment of this disease which have recently been established, as well as those older establishments which have kept pace with the progress of knowledge, manual labour, in many of its forms, amusements, and sources of literary and scientific entertainment and instruction, have been introduced among the patients. In short, instead of being degraded to a level not only with criminals, but with the brute creation, and consequently shut out from association with mankind, and placed beyond the influence of kindness and of sympathy, the insane are now treated as intelligent and immortal beings, the affections and sympathies of whose hearts are still alive to the influences which operate upon those of mankind in general.

*Labour.*—"We have seen," says one of the reports of the M'Lean Asylum, Massachusetts "the very best results from labour. Patients who, without it, were noisy and troublesome, have become quiet with it. One patient, who was brought to the institution in irons, and who, until employed, was constantly raving and excited, when furnished with occupation became quiet." At the Massachusetts State Lunatic Hospital, in 1839, no less than one hundred and seventy-nine patients were employed in manual labor. The superintendent of that institution says, in his seventh report: "Of the benefit of labour, both for the curable and incurable insane, we have been long impressed. It promotes health, induces sleep, favours self-control, satisfies the individual of the confidence reposed in him by the officers of the institution, and produces quiet and contentment."

At the Pennsylvania Hospital, the Asylums at Frankford, New York, and Hartford, and at the state institutions of Maine, Vermont, Maryland, Virginia, and Ohio, the patients are induced to labour; and testimonies in favour of the utility of the practice might be adduced from nearly all of them.

In Europe similar sentiments prevail. "As employment," says Sir Andrew Halliday, speaking of the patients of the Armagh Asylum, Ireland, "is now generally allowed to be the best restorative, every means has been used to promote it. Such as are at all capable among the females are constantly employed in plain work, spinning, &c.; and the division in which this is going on is remarkable for its regularity and cheerfulness." At the Middlesex County Lunatic Asylum, at Hanwell, England, in 1837, of six hundred patients, more than four hundred were constantly engaged in some useful occupation.

In reference to this element of moral treatment, Samuel Tuke remarks, "The employment should, as far as it is practicable, be adapted to their (the patients') previous habits, inclinations, and capacities." He prefers that in which the individuals will excel, and the useful rather than amusing, as affording to the patient "a calm feeling of satisfaction." "It is related," says the same author, "of an institution in Spain, which accommodated all ranks, and in which the lower classes were usually employed, that a great proportion of these recovered, while the number of grandees (that recovered) was exceedingly small."

M. Brierre de Boismont, in a recent work, speaking of the patients at the Bicêtre, Paris, says, "The convalescent maniacs have this year excavated large quantities of earth; they have engaged with pleasure in farming, and have kept a laundry in operation. More than one hundred and fifty of them are employed in throwing up terraces, in masonry, gardening, the manufacture of locks, making plaster, cabinet-making, and carpentry." Dr. Ferrus, who has long had the medical charge of the patients in the Bicêtre, in speaking of labour, says, "I have made, myself, on a large scale, a happy experiment of its efficacy as a means of both discipline and cure. The recoveries have been more rapid, and the relapses more rare." Similar testimony might be adduced from the Asylum of Sonnenstein in Prussia, from those of Turin and Milan, in Italy, and from various institutions of the kind in France and Great Britain.

It is a remarkable fact, that, although farming implements and edged tools have, for many years, been entrusted to the insane, there is not a single instance on record of any serious injury having arisen therefrom.

*Amusements.*—It is universally acknowledged that suitable amusements are efficacious adjuvants in restoring the excited and deluded minds of the insane to their healthy standard of calmness and accuracy of perception. Hence, in nearly every asylum, whether foreign or domestic, the means are furnished by which the patients may engage in a diversity of games. Entertainment is afforded by an occasional tea-party or dancing party, and the means of intellectual gratification and instruction are supplied by books, newspapers, and magazines. Those patients who have a particular taste or predilection for any special science, or in whom such taste can be awakened, ought to be supplied with the means of pursuing it. At the Frankford Asylum, during the past year, a gentleman who had been several months under medical treatment, became, by an incidental circumstances, interested in botany. He immediately commenced the study of it, devoting himself with the most untiring assiduity to the pursuit. Books, and a microscope to facilitate in the analysis of flowers, were furnished him, and he was permitted to ramble alone through the woods and fields for the purpose of collecting specimens. He improved very rapidly in both physical and mental health, and soon returned to his home perfectly restored.

Music has been tried as a curative means in many Asylums. We should suppose, *a priori*, that it might be attended with beneficial results. In order, however, that this should obtain, it must be managed with a most discriminating judgment. It must be adapted to each patient, according as he is depressed or exalted; otherwise, the melancholy in the former case might be augmented, and the exaltation, in the latter, increased to fury. Esquirol, whose experience in this respect is undoubtedly greater than that of any other person now living, remarks, "I have tried it (music) in every manner, and under circumstances the most favourable to success. Sometimes *it has irritated the patient even to fury*; often it has tended to divert the attention, but *I cannot say that it has contributed to a cure.* It has been advantageous to the convalescent." * * *

"Having made so many partial applications of music, I was desirous of attempting it upon many persons, simultaneously. My experiments were made during the summer of 1824, and that of 1825. Many distinguished musicians of the capital, seconded by the students of the Conservatory of Music, assembled at our Asylum (La Salpêtrière) many Sabbath-days in succession. The harp, the piano, the violin, some wind instruments, and some excellent voices, combined to render our concert as agreeable as interesting.

"Eighty insane women, chosen by me from the convalescents, the maniacs, the tranquil monomaniacs, and some lypemaniacs, were commodiously seated, facing the musicians, in the dormitory of the convalescents. * * * * Airs of all kinds, of all metres, and upon all keys, were played and sung, varying the number and the nature of the instruments. Some great pieces of music were also executed. My patients were very attentive, their countenances became animated, the eyes of many beamed with additional brilliancy, but all remained tranquil. Some tears were shed. Two of the patients asked permission to sing an air, and to be accompanied: the request was granted.

"This novel spectacle was not without influence upon our unhappy patients, but we obtained no cure, *not even an amelioration of their mental condition.* After these concerts, each of which lasted two hours, the musicians went into the apartment among the patients, and executed, upon wind instruments, various well-known popular airs, of a martial or sentimental character. A great number of the women became excited, exalted at the sound of the instruments, and many, among the furious, formed circles in order to dance. This excitement was transient, and passed off almost as soon as the music ceased."

After some further observations, the author finally concludes, "If music does not cure, it diverts, and consequently soothes. It produces some alleviation, both physical and moral; it is evidently useful to the convalescent, and consequently it is not necessary to discontinue its use."

The human mind is prone to extremes, as the musical chord, which, if deviated from the line of tension, recoils to nearly the same extent upon the opposite side. And, to pursue the simile, the harmony of the former, like that of the latter, is the most agreeable when those extremes are least. The most intimate friend, when once estranged, is liable to become the most implacable enemy. Some physicians. discarding the antiphlogistic, adopt the ever stimulant method of treatment; and others, once acccustomed to drugging their patients to a most liberal extent, reject this practice and adopt the infinitessimal principles of homœopathy. Governments fall from despotism into anarchy, and from anarchy return to despotism. In the modern laudable crusade against distilled spirits, some of the leaders in the cause, the hermit-Peters of the warfare, have banished not only spirits, wine, and fermented liquors, but tea, coffee, meat, and condiments, by their code of dietetics. In all these instances, perhaps, there is a "golden mean," which is better and wiser than either extreme. In the moral treatment of insanity care is required, lest, in the recoil from the barbarity of former times, the opposite error be not avoided.

These remarks have been suggested by the account given by Esquirol of the introduction of theatrical entertainments at Charenton. This was done about the year 1805, and continued until 1811. A theatre was constructed, comedies, operas, and dramas were enacted, and, occasionally, fire-works were displayed. "Every body was pleased with it; great and small, wise and ignorant, were desirous of being at the exhibition given by the lunatics of Charenton. All Paris flocked there for several years; some from curiosity, others to judge of the prodigious effects of this admirable means of curing the insane. The truth is, this means effected no cure. * * * Those who were to witness the exhibition were selected by favour. This excited jealousies, quarrels, and bitterness of feeling. Hence occurred sudden explosions of delirium and returns of mania and of fury. * * * That which passed at Charenton teaches us sufficiently upon this subject. How many were the relapses, how numerous the paroxysms of fury, provoked by these theatrical representations! Never have we seen patients cured by this mode of treatment."

Since the foregoing remarks were written, a treatise upon Insanity has appeared in Paris, the object of which is "to establish the truth of the following three propositions:—

1. If it be true that insanity depends on an alteration, or morbid condition of the encephalon, we are completely ignorant in what this alteration consists.

2. The moral treatment of the insane, as usually practised, is considered only as an auxiliary of the physical treatment.

3. In the insane, the intellect and passions cannot be brought back to their healthy type, or standard, without the aid of moral treatment; and this mode of treatment is the only one which has a direct influence on the symptoms of insanity."*

The author of this work is a highly intellectual and scentific physician, and his experience in the treatment of the insane has been very extensive; hence his opinions merit attention and respect. Those best acquainted with mental diseases, and with their present mode of treatment, will acknowledge an affirmative to the two first propositions, even without an investigation of the arguments adduced in the demonstration of the truth of them. But, it is believed that the same assent cannot be granted to the sentiment of the latter clause of the third proposition. Insanity is far from being an invariably idiopathic disease of the encephalon; it is frequently symptomatic of affections of the other viscera. Now, we would ask, what is that moral treatment, so potent in its influence as to correct the morbid conditions of the portal circle, or restore the dyspeptic stomach to its normal state? It is to be feared that this author has fallen into the extreme alluded to above.

*Attendants.*—How perfect soever may be an asylum in its organization and administration, how complete soever it may be in all the physical comforts which wealth may purchase or ingenuity invent, how diverse soever the means of recreation and amusement—the great object for which these have been furnished, that of effecting a cure in the patients, will be tardily, if indeed it be ever accomplished, unless the attendants, those who have the immediate care of those patients, are of a suitable character. Complete dominion over the passions, a well-cultivated mind, unyielding firmness, untiring energy, and an ever-watchfnl vigilance, united with mildness, gentleness, an affable and respectful deportment, and a benevolent, sympathising, Christian mind, are necessary to constitute the perfect attendant. The nearer this this standard be approached, the better will be the care extended to the patients, and the more rapid will be the cures. "We will not," says one of the reports of the M'Lean Asylum, "continue any male attendant, whom we cannot invite into our family, and seat at our table; and with whom we could not confidently place our wives, sisters, and brothers." The rule that immediate dismissal shall be the penalty of striking a patient, is adopted in most asylums, and should be in all. Could the standard which we have mentioned be reached, perhaps no such rule would be necessary.

*Coercion and Punishment.*—The strait-jacket, manacles, and chains have nearly been abandoned in the best institutions of the treatment of lunatics. The first, however, is still used occasionally in some of the British Asylums, and is not entirely abandoned in some of those of the United States. A change of location, the deprivation of a privilege, the shower-bath, and other punishments comparatively mild, are often sufficiently effective. The "tranquillizing chair," and mits and muffs, are still employed occasionally in nearly all asylums. In the Connecticut Retreat, as says one of the reports of that institution, "in case coercion and confinement become necessary, it is impressed upon his (the pationt's) mind, that this is not done for the purpose of punishment, but for his own safety and that of his keepers." "In no case," says the same report, "is deception of the patient employed or allowed; on the contrary, the greatest frankness, as well as kindness, forms a part of the moral treatment. His case is explained to him, and he is made to understand, as far as possible, the reasons why the treatment to which he is subjected has become necessary. By this course of intellectual management, it has been found, as a matter of experience, at our institution, that patients who had always been raving when confined without being told the reason, and refractory when commanded, instead of being entreated, soon became peaceful and docile." Sir A. Halliday insists upon the necessity of honourable and candid dealing with the insane, and

* Du Traitement Morale de la Folie. Par F. Leuret, Paris, 1840.

urges the importance of the fact, that they are generally, if not universally, affected by kindness, while they never forget injuries, insults, duplicity, or imposition. An appeal to the sympathies of the most maniacal patients, while, at the same time, a negative assent is given to their particuliar hallucination, is sometimes more effective than punishment. An interesting instance of this kind is related by the late Dr. Rush, of a lunatic in the Pennsylvania Hospital. This patient having frequently attempted to set fire to the building, was expostulated with by one of the managers, who endeavoured to impress upon his mind the effects of a conflagration, such as he had attempted. "But I am a salamander," said he. "Recollect, however," answered the gentleman expostulating with him, "that all the patients in the hospital are not salamanders." This sagacious reply had the desired effect; the patient desisted from his incendiary attempts.

